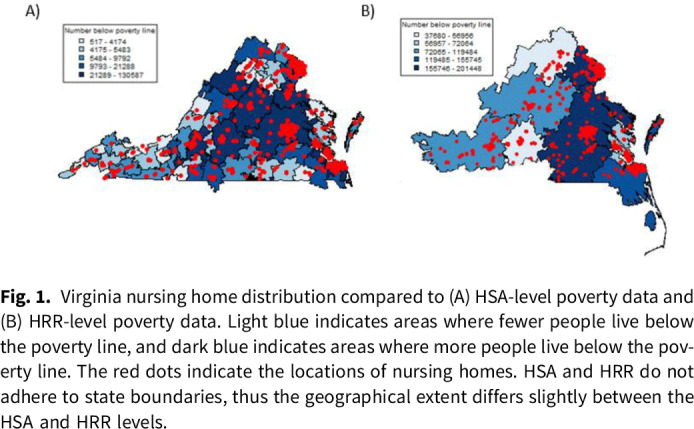# The geography of social vulnerability and nursing home facility factors related to infectious disease transmission

**DOI:** 10.1017/ash.2022.83

**Published:** 2022-05-16

## Abstract

**Background:** The impacts of health inequities on healthcare access, utilization, and outcomes have been highlighted by the COVID-19 pandemic, but these issues have been ongoing, yet understudied, in infectious disease epidemiology. Health inequities affect access to care, quality of care, and health outcomes in all healthcare settings. One healthcare setting that has yet to be fully studied in the context of health inequities is nursing homes. Nursing homes have a host of facility and population-specific issues that differ from other healthcare settings, making the impacts of health inequities likely unique and imperative to understand. The impacts of health inequities on nursing homes are unclear, and they likely have downstream effects on trends in morbidity, mortality, and transmission of multidrug-resistant organisms (MDROs) and other pathogens. **Method:** Here, we present a descriptive analysis, integrating multiple datasets relating to nursing home facility factors (data from the CMS Provider of Services and the CDC NHSN), nursing-home staffing trends (data from the CMS Payroll-Based Journal data), and social vulnerability (data from the CDC Social Vulnerability Index). We conducted a spatial analysis of nursing-home locations and the social vulnerability of the area. **Results:** Investigations of facilities and health inequities are best conducted in small spatial geographies. Analyses with less detailed spatial geographies miss high levels of heterogeneity in social vulnerability. Figure 1 provides an example, showing that analyzing nursing homes at a smaller spatial scale (ie, healthcare service area or HSA) shows heterogeneity in poverty levels that might be overlooked at a rough spatial scale, like Hospital Referral Regions (HRR). The poverty level associated with a nursing home will differ greatly depending on the geography of the analysis. **Conclusions:** These findings highlight that health inequities affect the quality and quantity of care of in nursing homes and that research conducted at larger geographical scales may overlook important mechanistic factors. This work will inform epidemiological models for disease transmission in nursing homes, accounting for the impacts of health inequities on transmission. Abating health inequities in all healthcare settings is a necessity to improve public health for the entire United States.

**Funding:** None

**Disclosures:** None

**Figure:**